# Effect of Blood Pressure Control on Cardiovascular Events in Patients with Chronic Kidney Disease: A Systematic Review

**DOI:** 10.7759/cureus.86230

**Published:** 2025-06-17

**Authors:** Anas E Ahmed, Khaled W Halawany, Faizah S Alyahyawi, Abdullah H Khormi, Hussain M AlQibti, Othman M Saifain, Talal M Alqarni, Jana S Alqurashi, Rose M Alabdali, Shoog T Alowaimer

**Affiliations:** 1 Community Medicine, Jazan University, Jazan, SAU; 2 Pharmacy, Armed Forces Hospital, Wadi Al Dawasir, Riyadh, SAU; 3 Internal Medicine, King Fahad Central Hospital, Jazan, SAU; 4 Internal Medicine, King Fahad Central Hospital, Bisha, SAU; 5 College of Medicine, University of Bisha, Bisha, SAU; 6 College of Medicine, Taif University, Taif, SAU

**Keywords:** acute kidney injury, antioxidant therapy, blood pressure control, cardiovascular disease, cardiovascular outcomes, chronic kidney disease, hypertension, nutraceuticals, renin-angiotensin-aldosterone system blockers, sodium-glucose cotransporter inhibitors

## Abstract

Cardiovascular disease remains the leading cause of morbidity and mortality in patients with chronic kidney disease (CKD), with hypertension, common in this population, contributing significantly to vascular damage and cardiovascular risk. This systematic review evaluates the impact of blood pressure (BP) control on cardiovascular outcomes in individuals with CKD. A comprehensive search of PubMed, Cochrane Central Register of Controlled Trials (CENTRAL), Scopus, and Web of Science up to May 5, 2025, identified randomized controlled trials (RCTs) and prospective comparative studies assessing BP interventions and cardiovascular outcomes in CKD populations. Methodological quality was appraised using the Modified Downs and Black checklist. Of the 11,606 studies screened, 10 met the inclusion criteria. Interventions included intensive BP targets, renin-angiotensin-aldosterone system (RAAS) blockers, sodium-glucose cotransporter-2 (SGLT2) inhibitors, and antioxidant therapies. Most studies reported significant reductions in cardiovascular events, particularly with intensive BP control, though risks such as acute kidney injury (AKI) and hyperkalemia were noted. Nutraceuticals showed potential for anti-inflammatory and BP-lowering benefits. Overall, the methodological quality was high, with most studies rated as good to excellent. Targeted BP control appears to significantly reduce cardiovascular risk in CKD patients; however, individualized treatment strategies are essential to minimize adverse renal outcomes. While the evidence supports the cardiovascular benefits of BP management in this population, further research is needed to optimize intervention strategies and define safety thresholds.

## Introduction and background

Chronic kidney disease (CKD) affects approximately 10% of the global population and is closely linked to increased cardiovascular morbidity and mortality [1,2]. Cardiovascular disease is the leading cause of death in this group, as patients with CKD face a significantly higher risk of cardiovascular events compared to the general population [3,4]. The decline in renal function adversely affects vascular health, necessitating proactive strategies to reduce cardiovascular risk.

Hypertension, present in most individuals with CKD, is both a cause and consequence of the disease [5,6]. Poor blood pressure control (BP) accelerates kidney damage and contributes to vascular remodeling, atherosclerosis, and left ventricular hypertrophy, key drivers of cardiovascular events [7,8]. Consequently, BP management is a central focus of CKD care guidelines, with recommendations tailored to disease stage and comorbidities. According to the most recent Kidney Disease: Improving Global Outcomes (KDIGO) guidelines, the comprehensive management of CKD involves three therapeutic pillars: BP control, renin-angiotensin-aldosterone system (RAAS) inhibition using angiotensin-converting enzyme (ACE) inhibitors or angiotensin II receptor blockers (ARBs), and reduction of proteinuria. ACE inhibitors and ARBs play a foundational role, particularly in patients with albuminuria, due to their dual benefits in lowering BP and mitigating glomerular injury, thereby slowing CKD progression and reducing cardiovascular risk [1-5]. 

Large clinical trials have shown that lowering systolic BP reduces cardiovascular events in high-risk groups [9,10]. However, the ideal target in CKD remains debated due to potential adverse effects, especially with intensive control strategies. According to the 2021 KDIGO guidelines, a target systolic BP of <120 mmHg is recommended for most patients with CKD (not on dialysis), provided it can be achieved safely, using standardized office BP measurements [11].

Various interventions are used to manage hypertension in CKD, including RAAS inhibitors, sodium-glucose co-transporter-2 (SGLT2) inhibitors, and lifestyle changes [12,13]. Their efficacy and safety can differ based on CKD stage, comorbidities, and treatment intensity, highlighting the need for individualized approaches.

This systematic review aims to evaluate and synthesize evidence from randomized controlled trials (RCTs) and prospective studies on the impact of BP control on cardiovascular outcomes in CKD. It also identifies gaps in the literature to guide future research.

## Review

Methods

Literature Search Strategy

This systematic review was conducted and reported following the Preferred Reporting Items for Systematic Reviews and Meta-Analyses (PRISMA) guidelines [14]. A comprehensive search was performed in PubMed, Cochrane Central Register of Controlled Trials (CENTRAL), Scopus, and Web of Science, covering all studies published up to May 5, 2025. The strategy used controlled vocabulary (e.g., MeSH terms) and free-text keywords combined with Boolean operators to maximize sensitivity and relevance. The PubMed search string included: (“Hypertension”[Mesh] OR “Blood Pressure”[Mesh] OR “blood pressure control” OR “blood pressure lowering” OR antihypertensive OR “intensive blood pressure control”) AND (“Kidney Diseases”[Mesh] OR “Chronic Kidney Disease” OR CKD OR “renal insufficiency” OR “end-stage renal disease” OR ESRD) AND (“Cardiovascular Diseases”[Mesh] OR “Cardiovascular Events” OR “Heart Failure” OR “Myocardial Infarction” OR “Stroke” OR “Cardiovascular mortality”). Equivalent terms were adapted for other databases. Filters limited results to human adults and English-language publications. Reference lists of the included studies were also manually screened for additional relevant articles by two independent reviewers. Any disagreements were resolved through discussion or consultation with a third reviewer.

Eligibility Criteria

Studies were selected based on the PICO framework (Population, Intervention, Comparison, Outcome, Study design) [15]. We included RCTs and prospective comparative studies, published in English, that enrolled adult patients with CKD stages 3 to 5 or those undergoing dialysis. Interventions comprised any BP control strategy, pharmacological, target-based (e.g., intensive systolic BP control), or non-pharmacological. Comparators included usual care, placebo, or alternative antihypertensive regimens. Studies needed to report at least one cardiovascular outcome such as myocardial infarction, heart failure, stroke, cardiovascular mortality, or validated surrogate markers like arterial stiffness or left ventricular mass index. However, we acknowledge that our initial search string did not explicitly include these terms, which may have limited the identification of studies primarily focused on these surrogate endpoints. Exclusions included case reports, case series, reviews, editorials, conference abstracts, trial registrations, animal studies, and non-English publications.

Data Extraction

Full texts of 10 studies meeting the inclusion criteria were reviewed. Extracted data included study design, country, sample size, CKD stage, BP intervention details (drug class, dose, duration), comparator groups, outcome measures (cardiovascular events, mortality, surrogate markers), and key findings. Any discrepancies were resolved through reviewer discussion.

Quality Appraisal

Methodological quality was assessed independently by two reviewers using the Modified Downs and Black checklist [16], which evaluates 27 items across five domains: reporting, external validity, internal validity (bias and confounding), and statistical power. Studies were classified as excellent (26-28 points), good (20-25 points), fair (15-19 points), or poor (≤14 points). Any scoring disagreements were resolved by consensus.

Results

Study Selection

**Figure 1:**
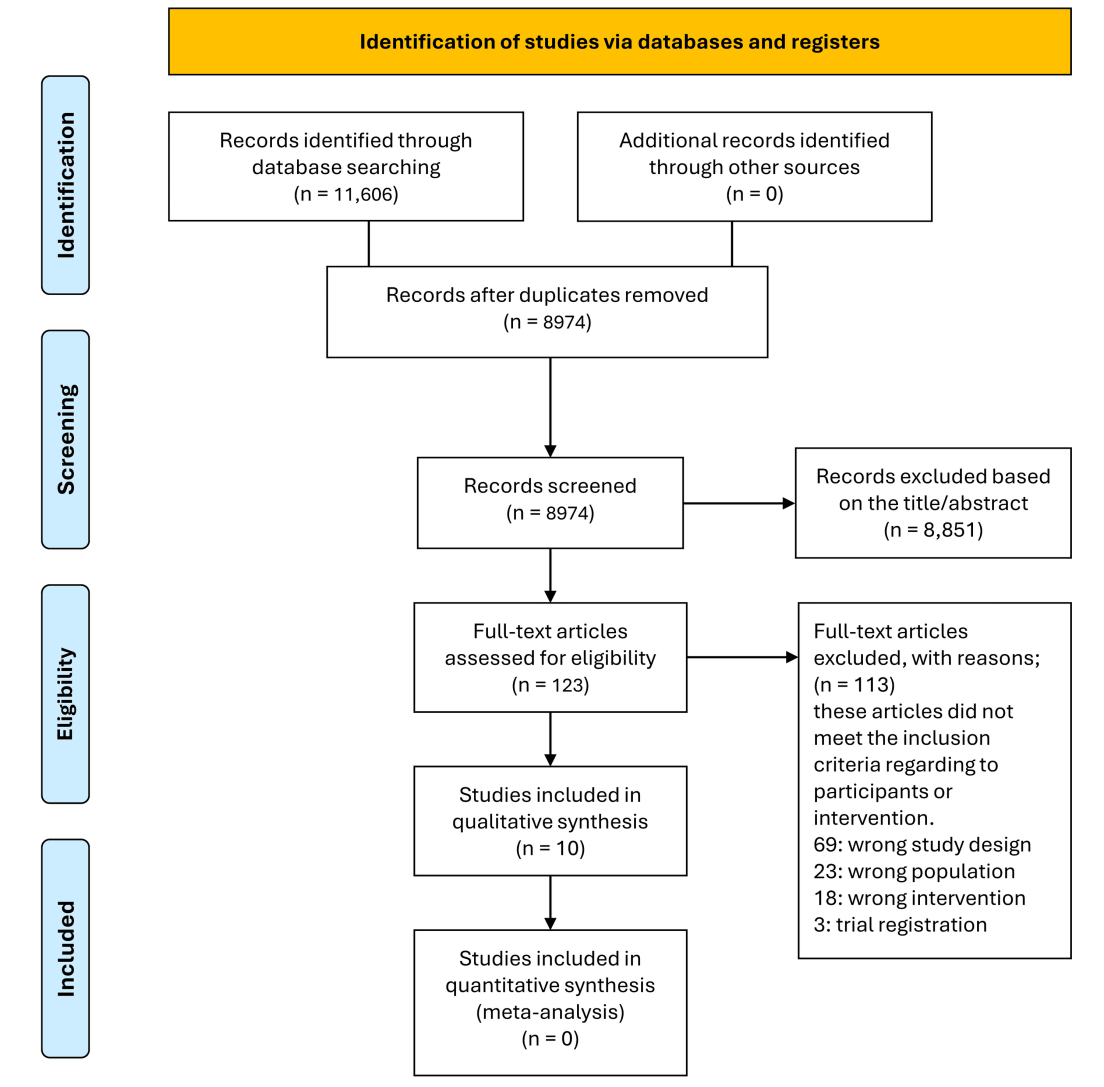
PRISMA flowchart for the search and selection of the studies

The systematic search identified 11,606 records from PubMed (1,475), Cochrane Library (2,646), Scopus (2,980), and Web of Science (4,505). After removing duplicates, 8,974 records were screened by title and abstract, and 8,851 were excluded as being irrelevant. Of the 123 full-text articles assessed, 113 were excluded for not meeting the inclusion criteria. Ten studies [17-26] were included in the qualitative synthesis. The PRISMA flow diagram details the selection process (Figure 1).

Study Characteristics

Studies were conducted across various countries, including Germany [17], Canada [18], Iran [19], the UK [20], Italy [22], Thailand [23], and the US [21,24-26]. Most were RCTs with double-blind, placebo-controlled, or crossover designs. Two were secondary analyses of the Systolic Blood Pressure Intervention Trial (SPRINT) trial [21,26]. Sample sizes ranged from 16 to over 9,000 participants. Participants were adults with CKD stages 3-5 or end-stage renal disease (ESRD) receiving dialysis, aged approximately 50 to 72 years, with a slight male predominance. Although some included studies reported participants with earlier stages of CKD (stages 1-2), the eligibility criteria for this review were restricted to stages 3-5 and dialysis-dependent ESRD. Stratification by kidney function (estimated glomerular filtration rate (eGFR), albuminuria) was common in subgroup analyses (Table 1).

**Table 1:** Summary of studies included in the review This table summarizes the characteristics and findings of included studies. RCT, Randomized Controlled Trial; CKD, Chronic Kidney Disease; ESRD, End-Stage Renal Disease; HF, Heart Failure; CV, Cardiovascular; CVD, Cardiovascular Disease; BP, Blood Pressure; CRP, C-reactive protein; eGFR, estimated Glomerular Filtration Rate; ACR/uACR, Urinary Albumin-to-Creatinine Ratio; SBP/DBP, Systolic/Diastolic Blood Pressure; LVMi, Left Ventricular Mass index; LVEF, Left Ventricular Ejection Fraction; NYHA, New York Heart Association class; CVD, Cardiovascular Disease; SAEs, Serious Adverse Events; IMT, Intima Media Thickness; CIMT, Carotid Intima-Media Thickness; PON-1, Paraoxonase-1; PSH, Protein Sulfhydryl; TAC, Total Antioxidant Capacity; MDA, Malondialdehyde; IL-6, Interleukin-6; hsCRP, high-sensitivity C-Reactive Protein; PWV, Pulse Wave Velocity; cfPWV, carotid-femoral PWV; CAC, Coronary Artery Calcification; CAVI, Cardio-Ankle Vascular Index; HR, Hazard Ratio; NS, Not Significant.

Author(s), Year	Study design	Country	Participants	CKD stage/type	Intervention	Comparator	Outcomes	Results
Hammer et al., 2019 [17]	RCT (Double-blind, placebo-controlled)	Germany	97 patients (50 Spironolactone, 47 Placebo); Mean age 60.3 ± 13.2 yrs	ESRD on hemodialysis	Spironolactone 50 mg daily for 40 weeks	Placebo daily for 40 weeks	Primary: LVMi change; Secondary: BP, LVEF, walk test, NYHA class, hyperkalemia, renal function	No significant LVMi change; hyperkalemia more frequent in treatment group; 0 deaths in treatment vs 4 in placebo group
Al Hamarneh et al., 2017 [18]	Subgroup analysis of RCT (RxEACH)	Canada	290 CKD patients (147 intervention, 143 control); Mean age ~61 yrs	eGFR <60 or ACR ≥3 mg/mmol	Pharmacist-led CV risk management	Usual care	CV risk score, BP, LDL, HbA1c, smoking cessation, medication use	CV risk ↓20%, SBP ↓10.5 mmHg, LDL ↓0.2 mmol/L, HbA1c ↓0.7%; more med adjustments in intervention
Barati Boldaji et al., 2020 [19]	Randomized crossover trial	Iran	41 ESRD patients on hemodialysis; Age 24–65 yrs	ESRD on hemodialysis	Pomegranate juice 100 mL 3×/week for 8 weeks	Usual care (crossover)	BP, lipids, IL-6, MDA, TAC	SBP/DBP ↓; IL-6 & MDA ↓; HDL & TAC ↑; LDL unchanged
Ng et al., 2016 [20]	Feasibility RCT (Double-blind, placebo-controlled)	UK	16 randomized; Target CKD stage 3	Stage 3 CKD, non-diabetic	Spironolactone 25 mg daily for 40 weeks	Placebo	Arterial stiffness (PWV), hyperkalemia, renal function	Trial terminated early due to poor recruitment; feasibility data only
Cheung et al., 2017 [21]	RCT (SPRINT subgroup analysis)	United States	2646 CKD patients; Mean age 72 yrs	Stage 3 CKD (eGFR 20–59), non-diabetic	Intensive SBP target <120 mmHg	Standard SBP <140 mmHg	CVD events, mortality, eGFR decline, SAEs	HR for CVD 0.81; all-cause death HR 0.72; more renal decline and hyperkalemia in intensive group
Zinellu et al., 2016 [22]	RCT	Italy	24 patients with stage 3–4 CKD; Mean age 60 yrs	Stage 3–4 proteinuric CKD	Telmisartan + Ramipril for 6 months	Telmisartan alone	PSH, IMT, BP, proteinuria	IMT ↓, BP ↓, PSH ↑ with combination therapy; no GFR change
Mayne et al., 2024 [25]	RCT (Double-blind, EMPA-KIDNEY substudy)	UK & Germany	660 CKD patients; Mean age 64 yrs	eGFR ≥20 to <45, or 45–90 with uACR ≥200 mg/g	Empagliflozin 10 mg daily for 18 months	Placebo	Fluid overload (bioimpedance), CV events, weight, BP	Fluid overload ↓0.24 L; SBP ↓2.6 mmHg; CV composite HR 0.91 (NS)
Saengpanit et al., 2018 [23]	RCT (Open-label)	Thailand	50 ESRD patients on hemodialysis; Mean age ~52 yrs	ESRD on hemodialysis	Sodium thiosulfate IV 12.5 g twice weekly for 6 months	Usual care	CAVI, cfPWV, CAC, hsCRP, hemodynamics	CAVI ↓0.53, cfPWV ↓0.93 m/s, CAC stable; few mild side effects
Wu et al., 2015 [24]	RCT (Double-blind, placebo-controlled)	United States	33 ESRD patients on hemodialysis; Mean age ~54 yrs	ESRD on hemodialysis	Pomegranate extract 1000 mg daily for 6 months	Placebo	BP, CIMT, PWV, CRP, IL-6, PON-1 activity, physical function	SBP ↓24 mmHg, DBP ↓10 mmHg (NS after adjustment); lactonase ↑27%; other outcomes unchanged
Vaduganathan et al., 2020 [26]	Post hoc analysis of RCT (SPRINT-HF substudy)	United States	9361 participants (28.4% with eGFR <60); Mean age 66–72 yrs	CKD stages 1–3 (excluded if eGFR <20 or proteinuria >1 g/day)	Intensive SBP <120 mmHg	Standard SBP <140 mmHg	HF events, HF/CV death	HF risk ↑ with lower eGFR and higher UACR; HR for HF in eGFR <60 & UACR >300 = 8.40; treatment effect consistent (P> 0.05)

Interventions included pharmacological agents such as spironolactone [17,20], telmisartan plus ramipril [22], and empagliflozin [25], alongside non-pharmacological approaches like pomegranate supplementation [19,24], intravenous sodium thiosulfate [26], and pharmacist-led cardiovascular risk management [18]. Comparators were standard care or placebo; SPRINT studies compared intensive versus standard BP targets. Outcomes covered cardiovascular events, vascular and renal surrogate markers, BP, and inflammatory markers.

Quality Assessment

As per the Modified Downs and Black checklist, study quality was generally high. Most studies scored well on reporting (up to 10/10) and internal validity (up to 7/7). External validity varied, with lower scores in small or single-center trials [19,22,24]. Some studies had limitations such as early termination [20] or lack of power calculations [19,24]. The highest quality scores (27/28) were observed in three studies [18,21,25]. Overall evidence quality was strong with minor variability due to study size and setting (Table 2).

**Table 2:** Quality assessment of the included studies This table presents the quality assessment of studies included in the review. The assessment was conducted using the Downs and Black checklist [16], a validated tool designed for evaluating both randomized and non-randomized studies of healthcare interventions.

Study	Reporting	External validity	Internal validity – Bias	Internal validity – Confounding	Power	Total
Hammer et al., 2019 [17]	10	2	6	6	1	25
Al Hamarneh et al., 2017 [18]	10	3	7	6	1	27
Barati Boldaji et al., 2020 [19]	10	2	6	5	1	24
Ng et al., 2016 [20]	9	2	5	5	0	21
Cheung et al., 2017 [21]	10	3	7	6	1	27
Zinellu et al., 2016 [22]	10	2	6	5	1	24
Mayne et al., 2024 [25]	10	3	7	6	1	27
Saengpanit et al., 2018 [23]	9	2	6	5	1	23
Wu et al., 2015 [24]	9	2	6	5	0	22
Vaduganathan et al., 2020 [26]	10	3	7	6	1	27

Effect of Interventions

Intensive systolic BP control (<120 mmHg) reduced all-cause mortality (hazard ratio or HR 0.72) and showed a non-significant trend toward fewer cardiovascular events [21]. Spironolactone showed no benefit on left ventricular mass or ejection fraction in ESRD [17]. Combination of telmisartan and ramipril reduced carotid intima-media thickness [19]. Sodium thiosulfate decreased arterial stiffness and stabilized coronary calcification [26].

Systolic BP reductions were largest in intensive control (−12.3 mmHg) [21] and pomegranate juice (−10.2 mmHg) groups [19]. Pomegranate extract initially lowered systolic and diastolic BP but this lost significance after adjustment [21]. Telmisartan/ramipril significantly reduced BP; monotherapy did not [22]. Empagliflozin caused modest BP reductions [25], pharmacist-led interventions improved systolic BP [18], whereas spironolactone showed no significant BP changes [17,20].

Intensive BP control increased rapid renal decline but not ESRD or severe estimated glomerular filtration rate (eGFR) deterioration [21]. Empagliflozin reduced extracellular fluid overload and loop diuretic use [25]. Sodium thiosulfate did not affect calcium, phosphate, or parathyroid hormone (PTH) levels [26]. RAAS therapies and spironolactone did not significantly change creatinine, proteinuria, urine volume, or GFR [17,22].

Pomegranate supplementation lowered inflammatory and oxidative stress markers IL-6 and malondialdehyde (MDA), increased high-density lipoprotein (HDL) and antioxidant capacity [19]. Pomegranate extract raised antioxidant enzyme activity but did not affect CRP or other markers [24]. Sodium thiosulfate showed a non-significant high-sensitivity CRP decrease [26], empagliflozin improved HbA1c and hematocrit [25], and pharmacist-led care improved glycemic and lipid profiles [18].

Safety profiles varied: intensive BP control increased risks of acute renal failure, hyperkalemia, and hypotension [21], whereas spironolactone increased moderate hyperkalemia events [17]. RAAS combination therapy and sodium thiosulfate showed no serious safety issues [22,26]. Pomegranate treatments were well tolerated [16,21]. Similarly, empagliflozin and pharmacist-led interventions reported good adherence and a few adverse events [18,25].

Discussion

This systematic review of 10 studies demonstrates that targeted BP management in CKD patients is linked to improved cardiovascular outcomes, including reductions in myocardial infarction, heart failure, and cardiovascular mortality. Interventions focusing on intensive systolic BP control below conventional thresholds, whether pharmacological or structured approaches, showed the most pronounced benefits. These findings support the established understanding that effective BP control is crucial to mitigating cardiovascular risk in CKD populations. Most interventions were associated with reductions in systolic BP, with agents such as RAAS inhibitors, SGLT2 inhibitors, and dietary supplements like pomegranate extract demonstrating modest but statistically significant effects in individual studies. However, given the heterogeneity of study designs, populations, and outcome measures, a formal meta-analysis was not performed, limiting the ability to quantitatively synthesize these findings.

Renal outcomes were more variable, with some studies reporting stable or improved renal markers, while others raised concerns about accelerated decline in kidney function or acute kidney injury following intensive BP lowering. This highlights the ongoing clinical challenge of balancing cardiovascular benefits with potential renal risks in CKD management. Additionally, some studies suggested a potential link between BP control and reductions in inflammatory and oxidative stress markers, though evidence remains inconsistent. Safety profiles varied among interventions, with spironolactone and intensive BP reduction associated with higher rates of hyperkalemia and hypotension, underscoring the need for careful patient monitoring. Overall, these findings emphasize that individualized therapy, considering both cardiovascular and renal risks, is essential in optimizing BP management strategies for patients with CKD.

Further large-scale, long-term randomized controlled trials are essential to determine the optimal BP targets in CKD, particularly given the ongoing debate between intensive versus standard control strategies. Existing evidence often derives from heterogeneous populations and surrogate outcomes, limiting its generalizability. Moreover, few studies have stratified patients by CKD stage, presence of albuminuria, or dialysis status, despite the potential for differential responses to treatment. Future research should also aim to assess patient-centered outcomes such as cardiovascular mortality, progression to ESRD, and quality of life, while ensuring representation of key subgroups, including elderly patients and those with diabetic nephropathy. Additionally, trials incorporating newer antihypertensive agents, including SGLT2 inhibitors and mineralocorticoid receptor antagonists, could provide insights into synergistic or comparative effectiveness in various CKD stages.

A notable limitation of this review is the retrospective registration of the protocol on the International Prospective Register of Systematic Reviews (PROSPERO). Although the methodology was defined prior to data extraction and is transparently reported, the lack of prospective registration may reduce confidence in the review’s objectivity. We also acknowledge that restricting the search to English-language publications introduces a potential language bias, which could limit the comprehensiveness and generalizability of our findings. Additionally, while validated surrogate markers such as arterial stiffness and left ventricular mass index were considered eligible outcomes, our initial search strategy did not explicitly include terms for these markers. As a result, some relevant studies focusing on these endpoints may have been missed. To partially mitigate this, we manually screened the reference lists of included studies; however, the possibility of missing pertinent articles remains a limitation.

## Conclusions

This systematic review underscores the importance of targeted BP control in reducing cardiovascular risk among patients with CKD. Interventions, ranging from pharmacological therapies such as RAAS and SGLT2 inhibitors to non-traditional approaches like nutraceuticals, demonstrated varying degrees of effectiveness in lowering BP and improving cardiovascular outcomes. While intensive BP control is linked to reductions in cardiovascular events and mortality, it may increase the risk of renal adverse effects, particularly in advanced CKD. Overall, individualized BP management that balances cardiovascular benefits with renal safety is essential. Further large-scale, long-term studies are needed to establish optimal BP targets and identify the safest and most effective interventions across different stages of CKD.
